# Changes of Knee Phenotypes Following Osteotomy Around the Knee in Patients with Valgus or Varus Deformities—A Retrospective Cross-Sectional Study

**DOI:** 10.3390/jcm14134684

**Published:** 2025-07-02

**Authors:** Jennyfer A. Mitterer, Stephanie Huber, Matthias Pallamar, Sebastian Simon, Jan Nolte, Catharina Chiari, Jochen G. Hofstaetter

**Affiliations:** 1 Michael Ogon Laboratory for Orthopaedic Research, Orthopaedic Hospital Vienna Speising, 1130 Vienna, Austriaresearchlab@oss.at (J.G.H.); 2 Department of Paediatric Orthopaedics, Orthopaedic Hospital Vienna Speising, 1130 Vienna, Austria; 3 2nd Department, Orthopaedic Hospital Vienna Speising, 1130 Vienna, Austria

**Keywords:** osteotomy around the knee, coronal plane classification, knee phenotypes, artificial intelligence, lower limb alignment

## Abstract

**Background:** Osteotomies around the knee aim to correct varus or valgus malalignment and improve biomechanics. However, little is known about their effect on knee phenotypes, as defined by the Coronal-Plane-Alignment-of-the-Knee (CPAK) and Hirschmann’s functional classification. This study evaluated pre- and postoperative phenotypes in patients undergoing high-tibial-osteotomy (HTO) or distal-femoral-osteotomy (DFO). **Methods:** We retrospectively analysed 214 osteotomies around the knee (HTO: 145; DFO: 69) of 188 patients from our institutional registry. Radiographic parameters were measured using a validated artificial intelligence software, with phenotypes classified by CPAK and Hirschmann classification. Preoperative osteotomy planning was compared to postoperative alignment. Regression was used to assess the influence of demographic and radiographic factors. **Results:** CPAK types changed in 95.3% of cases. Medial opening HTOs most frequently shifted from CPAK type I (73.8%) to VI (42.3%), while medial closing DFOs transitioned from type III (81.5%) to V (24.1%). Concordance between planned and achieved CPAK types was highest for types III, IV, and V. Postoperative angles were generally smaller than planned for joint-line-obliquity (JLO), lateral-distal-femur-angle, and medial-proximal-tibial-angle (*p* < 0.001). Neutral JLO was restored in only 48.1%. Preoperative phenotypes NEUmLDFA0° (40.1%) and VARmMPTA3° (32.3%) were most common, while postoperative phenotypes included VALmLDFA3° (52.4%) and VALmMPTA3° (37.7%). Age, sex, and BMI significantly influenced alignment outcomes. **Conclusions:** Postoperative CPAK classifications shifted significantly across all osteotomy types, with minimal retention of preoperative types. Although most procedures achieved correction within the target HKA range, restoration of a neutral JLO was observed in only half of the cases, emphasizing the importance of phenotype-specific planning and highlight potential limitations of CPAK classification.

## 1. Introduction

Modern concepts of osteotomies around the knee (OAK) aim to correct lower limb malalignment and improve biomechanics in varus or valgus knees with unicompartmental osteoarthritis (OA) [1,2]. Alignment is conventionally assessed on standing long-leg radiographs (LLRs). Valgus malalignment is defined by a hip-knee-ankle angle (HKA) > 3° and a mechanical axis deviation (MAD) > 10 mm lateral to the knee joint centre, while varus malalignment is defined by an HKA < −3° and MAD > 15 mm medial to the knee joint centre [3,4,5]. Depending on the origin of the deformity, surgical options include high tibial osteotomy (HTO), distal femoral osteotomy (DFO), or double level osteotomies [6]. Previous studies recommend correction towards a distal femoral valgus of 3° and a proximal tibial varus of 3°, thereby achieving a neutral joint line obliquity (JLO) and rebalancing the load distribution between the medial and lateral compartments [1]. However, over- or under-correction may lead to excessive JLO, which is associated with poorer functional outcomes and reduced patient satisfaction [2,7].

Recent literature highlights a substantial variability in femoral and tibial phenotypes. The Coronal Plane Alignment of the Knee (CPAK) classification by MacDessi et al. [8] and the functional phenotype classification by Hirschmann et al. [9] reflect this anatomical diversity by incorporating constitutional limb alignment and supporting more personalised alignment strategies. The CPAK classification is intended for use in healthy and arthritic knees prior to knee arthroplasty and identifies knees which may benefit most from kinematic alignment when optimisation of soft tissue balancing is prioritized [8]. Huber et al. demonstrated gender-specific differences and a wide distribution of phenotypes in osteoarthritic knees prior to total knee arthroplasty (TKA) using both CPAK and phenotype classifications [10]. Furthermore, it has been shown that phenotype changes following TKA remain heterogeneous, despite the goal of achieving a mechanical alignment [11]. However, limited data exist on how osteotomies around the knee influence postoperative phenotypes, and it remains unclear whether changes towards a predominant postoperative CPAK type can be observed.

The aim of this study was to analyse pre- to postoperative changes in knee phenotypes in patients with valgus or varus deformity undergoing femoral or tibial osteotomy around the knee, using a validated artificial intelligence (AI) software. In addition, we evaluated the concordance between preoperative osteotomy planning and final postoperative radiographic outcomes.

## 2. Materials and Methods

### 2.1. Demographics

This study was approved by the ethics committee (EK: 46/2020). Between October 2009 and December 2021, we analysed 213 patients (127 male, 86 female) who underwent 253 monoplanar osteotomies around the knee from our prospectively maintained institutional osteotomy registry. Patients with congenital or metabolic deformities, post-traumatic deformities, hinge fractures, rotational or tibial tubercle osteotomies, prior arthroplasty, skeletal immaturity with open growth plates at the distal femur and/or proximal tibia, or missing calibration balls were excluded. Double-level osteotomies and medial or lateral opening DFOs were also excluded due to small subgroup sizes. A total of 214 osteotomies in 188 patients (male: 115; female: 73) were included in the final analysis. The median age was 42 years (range 14–65) and mean body mass index (BMI) was 27.1 ± 4.6 kg/m^2^. Preoperative alignment by HKA was varus in 136 (63.6%), neutral in 16 (7.4%), and valgus in 62 (30.0%) cases. Demographic details are in Table 1. All LLRs followed the hospital’s standard protocol with the patient standing and the patella facing forward, as per Paley and Pfeil [12].

### 2.2. Radiographic Assessment

Performed measurements included the hip-knee-ankle angle (HKA), mechanical axis deviation (MAD), mechanical lateral proximal femur angle (mLPFA), mechanical lateral distal femur angle (mLDFA), mechanical medial proximal tibia angle (mMPTA), mechanical lateral distal tibia angle (mLDTA), and joint line convergence angle (JLCA). Constitutional limb alignment was estimated by the arithmetic HKA (aHKA) as well as the joint line obliquity (JLO) and were calculated as follows: aHKA = mMPTA − mLDFA; JLO = mMPTA + mLDFA. Knees with an HKA > 3° were classified as valgus deformity, whereas HKA < −3° were considered as varus deformity [10]. Pre- and postoperative alignment sorted by HKA as well as pre- and postoperative radiographic measurements are displayed in Table 2 and Table 3.

The coronal plane alignment of the knee classification (CPAK) includes 9 types and uses a combination of the aHKA and JLO [8]. The groups classify the knee by the aHKA in neutral (aHKA 0° ± 2°), valgus (aHKA > 2°), or varus (aHKA < −2°) alignment, combined with the JLO with a neutral joint line (JLO 180° ± 3°), apex distal (JLO < 177°), or apex proximal JLO (JLO > 183°). As described before [10], the functional phenotype classification by Hirschmann et al. [9] was adapted, adjusting for mLDFA instead of mechanical medial distal femur angle (= 180° − mMDFA). Femur and tibia were classified into 5 types each by mLDFA and mMPTA with increments of 3° towards varus (VARmLDFA3°, VARmMPTA3°, VARmLDFA6°, VARmMPTA6°) or valgus alignment (VALmLDFA3°, VALmMPTA3°, VALmLDFA6°, VALmMPTA6°). The phenotype classification was analysed for HTO and DFO separately. In the HTO group, 13 (9.0%) preoperative and 17 (11.7%) postoperative tibiae exceeded the classification’s reference values and were therefore excluded. In the DFO group, there were 9 (3.3%) preoperative and 6 (2.8%) postoperative femurs that exceeded the classification’s reference values and were consecutively excluded from the analysis. Overall, 132/145 (91.0%) preoperative HTO, 128/145 (88.3%) postoperative HTO, 60/69 (87.0%) preoperative DFO, and 63/69 (91.3%) postoperative DFO were analysed for the phenotype-classification.

### 2.3. AI Software

We used AI Software IB Lab LAMA 1.03 (ImageBiopsy Lab™, Vienna, Austria) for automated measurements. The software had previously shown excellent reliability for LLRs after osteotomy (ICC: 0.81–0.99) [13]. Analysis took 5.7 h. All outputs were reviewed; 7 (3.3%) pre-op and 31 (14.5%) post-op measurements were remeasured manually using mediCAD^®^ v6.0 (Hectec GmbH, Landshut, Germany) by three orthopaedic surgeons (JAM, SH, SS). Preoperative planning was performed in mediCAD^®^ by each surgeon individually and retrospectively compared to the achieved postoperative alignment. The Fujisawa point was employed during preoperative planning, and postoperative target angles were set towards neutral JLO and HKA.

### 2.4. Statistical Analysis

Descriptive statistics used means ± standard deviations or frequencies. Data were stratified by deformity (varus, neutral, valgus) and osteotomy type (medial opening HTO, medial closing HTO, medial closing DFO, lateral closing DFO). CPAK changes were visualised using Sankey diagrams [14]. Chi-squared (χ^2^) tests compared categorical variables. Logistic regression assessed effects of gender, age, and BMI. Multiple linear regression evaluated HKA, mLDFA, mMPTA, aHKA, and JLO using the same covariates. R^2^ was used for model fit as per Cohen [15]. Statistical significance was considered for *p*-values < 0.05. The statistical analyses were performed with SPSS 25^®^ (IBM Corp. Released 2018. IBM SPSS Statistics for Windows, Version 25.0. Armonk, NY, USA), R Version 4.3.0, and GraphPad Prism 9 (GraphPad Software Inc, San Diego, CA, USA).

## 3. Results

### 3.1. Osteotomies

Preoperative analysis revealed CPAK type I as the predominant type in medial opening HTO, observed in 73.8% (96/130), transitioning postoperatively towards CPAK type VI in 42.3% (55/130). Retention of CPAK type was minimal (3.1%, 4/130) and primarily found in CPAK types I (2/4) and II (2/4). In medial closing HTOs, preoperatively CPAK type VI was mainly found (73.3%, 11/15) and predominantly transitioned to CPAK types II and III (40.0%, 6/15 each).

For medial closing DFOs, CPAK type III was the principally observed preoperative type (81.5%, 44/54), shifting postoperatively to CPAK type V in 11.1% (6/54). Concordance between pre- and postoperative CPAK types occurred in 9.3% (5/54), mainly for CPAK type III (4/5). In lateral closing DFOs, CPAK type IV was the most common preoperative type (53.3%, 8/15), transitioning postoperatively to CPAK type III in 80.0% (12/15). Retention of CPAK types in this group was observed only in one case of CPAK type IV (1/15, 6.7%).

**Figure 1:**
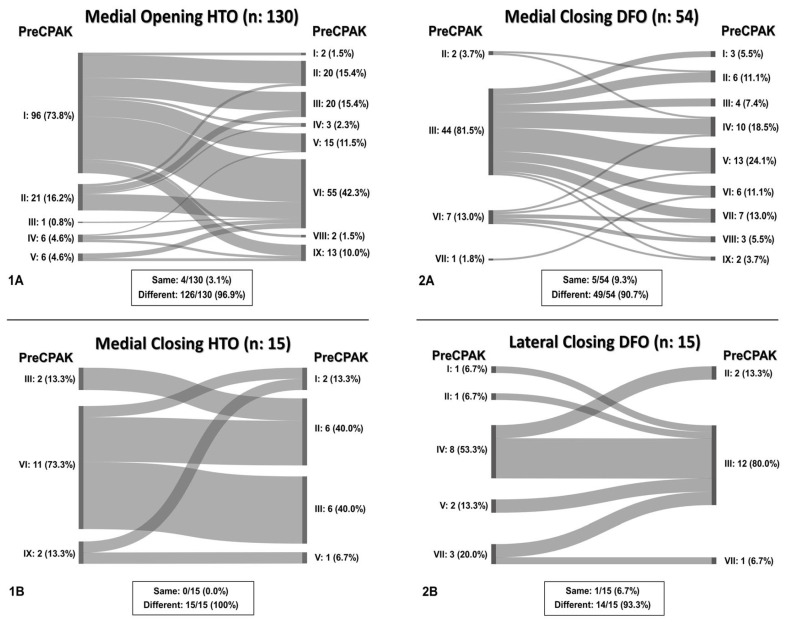
Sankey diagram displaying CPAK changes from pre- to post-operative types, grouped by osteotomy types. Numbers are absolute, frequencies in parentheses. (**1A**) Changes in CPAK types in the medial opening HTO group, (**1B**) changes in CPAK types in the medial closing HTO group, (**2A**) changes in CPAK types in the medial closing DFO group, (**2B**) changes in CPAK types in the lateral closing DFO group. Changes towards other CPAK types (“Different”) and persistence within the same CPAK type (“Same”) are displayed below the Sankey diagram. CPAK: coronal plane alignment of the knee, HTO: high tibial osteotomy, DFO: distal femoral osteotomy.

**Figure 2:**
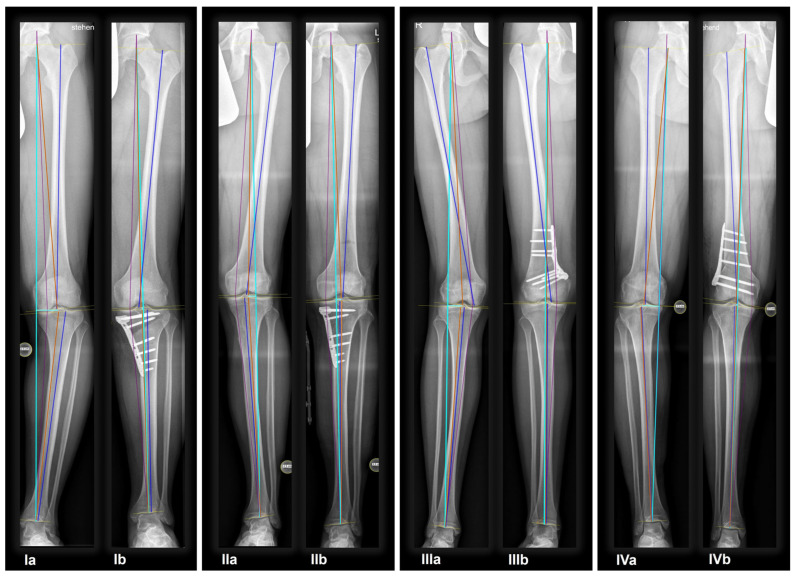
Example cases of phenotype changes. Mechanical femur axis (proximal orange line), distal femoral joint line (upper yellow line at the knee), proximal tibial joint line (lower yellow line at the knee), mechanical tibia axis (distal orange line), Mikulicz line (turquoise line) corresponding to the mechanical axis of the lower limb, mechanical-axis-deviation (horizontal turquoise line at the knee), femur length (purple line from the top of the femoral head to medial femoral condyle), and tibia length (purple line from medial femoral condyle to ankle). CPAK: coronal plane alignment of the knee, HTO: high tibial osteotomy, DFO: distal femoral osteotomy. HKA: hip-knee-ankle angle, MAD: mechanical axis deviation, mLDFA: mechanical lateral distal femoral angle, mMPTA: mechanical medial proximal tibial angle, JLCA: joint line convergence angle, aHKA: arithmetic HKA, JLO: joint line obliquity. **Ia/Ib:** Preoperative CPAK I to postoperative CPAK VI (medial opening HTO). *Ia—Preoperative:* HKA = −10.3°, MAD = 38.0 mm, mLDFA = 88.7°, mMPTA = 79.5°, JLCA = −1.1°, aHKA = −9.2°, JLO = 168.2°; *Ib—Postoperative:* HKA = 0.6°, MAD = −2.0 mm, mLDFA = 88.4°, mMPTA = 89.7°, JLCA = −1.7°, aHKA = 2.3°, JLO = 177.1°. **IIa/IIb:** Preoperative CPAK VI to postoperative CPAK II (medial closing HTO). *IIa—Preoperative:* HKA = 3.5°, MAD = −14.0 mm, mLDFA = 87.6°, mMPTA = 92.2°, JLCA = −1.2°, aHKA = 4.6°, JLO = 179.8°; *IIb—Postoperative:* HKA = −2.3°, MAD = 9.0 mm, mLDFA = 87.4°, mMPTA = 86.0°, JLCA = −0.8 °, aHKA = −1.4°, JLO = 173.4°. **IIIa/IIIb:** Preoperative CPAK III to postoperative CPAK V (medial closing DFO). *IIIa—Preoperative:* HKA = 6.3°, MAD = −23.0 mm, mLDFA = 82.2°, mMPTA = 90.9°, JLCA = −2.5°, aHKA = 8.7°, JLO = 173.1°; *IIIb—Postoperative:* HKA = −0.1°, MAD = 0.0 mm, mLDFA = 89.0°, mMPTA = 89.8°, JLCA = −0.8°, aHKA = 0.8°, JLO = 178.8°. **IVa/IVb:** Preoperative CPAK IV to postoperative CPAK III (lateral closing DFO). *IVa—Preoperative:* HKA = −9.1°, MAD = 36.0 mm, mLDFA = 92.1°, mMPTA = 86.6°, JLCA = −3.6°, aHKA = −5.5°, JLO = 178.7°; *IVb—Postoperative:* HKA = −0.9°, MAD = 3.0 mm, mLDFA = 84.3°, mMPTA = 86.4°, JLCA = −3.0°, aHKA = 2.1°, JLO = 170.7°.

Overall, 95.3% (204/214) of all osteotomies demonstrated a change in CPAK classification postoperatively. Preoperative varus alignment by HKA showed CPAK type I as the predominant type, while neutral preoperative alignment primarily featured CPAK type II as the most common preoperative classification. In contrast, preoperative valgus alignment by HKA revealed CPAK type III as the prevailing preoperative knee phenotype. There was a discernible overall shift, with CPAK VI becoming the predominant postoperative classification in the preoperative varus and neutral alignment by HKA at 36.1% (49/136) and 37.5% (6/16), while preoperative valgus by HKA showed a predominant transition to CPAK type V in 19.3% (12/62). Concordance between preoperative and postoperative CPAK types was highest for types IV and V (62.5% each), followed by type III (76.5%) and VI (51.0%). Concordance for other CPAK types ranged from 23.9% (type IX) to 50.0% (type II). Significant deviations between planned and achieved outcomes were noted in JLO, mLDFA, and mMPTA (*p* < 0.001), with mean differences of 2.2° for JLO and 1.1° for mLDFA and mMPTA. Postoperative angles were generally smaller than planned. Comprehensive information regarding the pre- to post-operative transition of CPAK classifications for the osteotomy types is presented in Figure 1 and Table 4. Figure 2 illustrates representative examples showcasing the most common changes from pre- to post-operative CPAK classifications.

### 3.2. Hirschmann’s Phenotype Classification

Preoperative phenotype analysis identified NEUmLDFA0° as the most common femoral phenotype (40.1%, 77/192) and VARmMPTA3° as the predominant tibial phenotype (32.3%, 62/192). The most frequent preoperative combination was NEUmLDFA0° with VARmMPTA3° (14.1%, 27/192). Conversely, rare preoperative combinations included VARmLDFA6° with VALmMPTA6° and VARmLDFA3° with VALmMPTA6° (0.5%, 1/192 each).

Postoperatively, VALmLDFA3° and VALmMPTA3° were the most frequent femoral and tibial phenotypes, observed in 52.4% (100/191) and 37.7% (72/191) of cases, respectively. The most common combination was VALmLDFA3° with VALmMPTA3° (17.3%, 33/191), while the rarest involved VALmLDFA3° with VARmMPTA6° (0.5%, 1/191). A detailed distribution of femoral and tibial phenotype classification is provided in Table 5.

### 3.3. Influences on Postoperative Radiographic Parameters

Logistic regression analysis revealed that female sex was associated with a higher likelihood of postoperative mLDFA outliers (OR = 1.94, *p* = 0.023). In medial opening HTOs, higher BMI significantly influenced postoperative JLCA (OR = 1.14, *p* = 0.012). Age was a significant predictor of postoperative HKA (OR = 1.07, *p* = 0.004), aHKA (OR = 1.06, *p* = 0.004), and mMPTA (OR = 1.08, *p* < 0.001). For medial closing DFOs, age influenced postoperative mLDFA (OR = 1.05, *p* = 0.038).

Linear regression demonstrated moderate predictive effects of gender and age on postoperative HKA in medial opening HTOs (gender: β = 1.30, *p* = 0.003; age: β = 0.07, *p* < 0.001; BMI: β = −0.002, *p* = 0.966; R^2^ = 0.148). JLCA was significantly predicted by age in medial closing DFOs (β = 0.07, *p* = 0.001, R^2^ = 0.188) and by age and BMI in medial opening HTOs (age: β = −0.04, *p* = 0.002; BMI: β = −0.09, *p* = 0.023; R^2^ = 0.145).

Postoperative aHKA was significantly influenced by age and BMI in medial opening HTOs (age: β = 0.12, *p* < 0.001; BMI: β = 0.09, *p* = 0.187; R^2^ = 0.227). Age had weak predictive value for JLO in medial opening HTOs (β = 0.10, *p* = 0.006; R^2^ = 0.108). Multiple regression for all osteotomies around knee showed moderate prediction of aHKA by age (β = 0.11, *p* < 0.001; R^2^ = 0.138).

## 4. Discussion

The main finding of this study was a heterogeneous distribution of knee phenotypes following osteotomies around the knee. While the vast majority achieved correction within the target HKA range (±3°), neutral JLO was reached in only 48.1%, and 95.3% of all osteotomies resulted in a change from the preoperative CPAK type. Changes within the osteotomy groups also showed high variabilities, where neutral joint line obliquity was achieved in about 50% in the medial opening HTO as well in the medial closing DFO group. In contrast, the medial closing HTO and lateral closing DFO tended to shift towards valgus orientation of the joint line. This finding supports the hypothesis that correction toward favourable joint line orientations may be highly influenced by multiple factors [8,9,10]. Micicoi et al. described three knee phenotypes (varus, neutral, valgus) using HKA and demonstrated a wide anatomical variability within these groups [6]. Our findings similarly indicate variability, but using the CPAK classification system offers a more nuanced view of the phenotype changes postoperatively. Notably, concordance between planned radiographic values and achieved postoperative values was highest for CPAK types IV and V, both at 62.5%, suggesting that these phenotypes might have a more predictable surgical outcome. Deviations between planned values and final achieved radiographic values for JLO, mLDFA, and mMPTA (*p* < 0.001) were relatively small but indicate a trend toward smaller postoperative angles than planned. This finding may be attributed to soft-tissue factors such as laxity or overcorrection, particularly in varus malalignments [16,17]. Additionally, in combined tibiofemoral deformities, severe malalignment, or in cases of additional ligament laxity, double-level osteotomies are considered the preferred surgical procedure [18].

In addition to using the Fujisawa point during preoperative planning, the intraoperative application of the ‘rule of thumb’ is an established method to ensure controlled deformity correction, estimate osteotomy height, reduce the risk of hinge fractures, and define a ‘safe zone’ for HTO [19]. However, in cases with a high preoperative JLCA, bony correction alone is often insufficient due to its correlation with soft-tissue laxity. In such cases, additional soft-tissue balancing is required [20]. All preoperative JLCA values were included in this study. The persistence of certain CPAK types postoperatively may be explained by variations in surgical technique or surgeon preference, particularly when soft-tissue balancing was not performed.

The heterogeneous shift in CPAK classifications observed postoperatively may be partially attributed to variability in surgical technique and surgeon-specific preferences, particularly regarding whether the osteotomy was performed on the femur or tibia. Studies have found that femoral osteotomy tends to achieve more reliable bony correction than tibial osteotomy [21,22]. As the CPAK classification simplifies femoral and tibial deformities based solely on limb alignment, it may not sufficiently account for individualized anatomical variations [23]. In cases of isolated femoral or tibial deformity, simplified classification systems such as CPAK may inadequately reflect patient-specific morphology and therefore have limited utility in osteotomy planning. This is particularly relevant in valgus malalignment, where the deformity may originate not only from the femur but also from the tibia or both segments combined [24].

Unlike the recommended postoperative femorotibial orientation of femoral 3° valgus and tibial 3° varus, we found that the predominating phenotypes were VALmLDFA3° and VALmMPTA3°. These discrepancies may result from overcorrection or soft-tissue imbalance. Mild valgus overcorrection in HTOs is sometimes intentional, particularly in varus knees [18]. Logistic regression revealed gender-specific differences in the alignment regarding the preoperative mLDFA. Intra-articular parameters such as the JLCA were significantly influenced by the BMI. Influences of demographic factors on preoperative intra- and extra-articular alignment parameters analysed by osteotomy type showed only weak associations. Gender-specific differences in knee phenotypes and the influence of patient-specific factors were addressed by Huber et al. recently, displaying significant associations between these demographic factors for HKA, JLCA, and mMPTA [10]. Age-dependent differences for patients undergoing osteotomy around the knee are widely discussed [25,26], whereby there seems to be no clear consensus whether age-specific differences in phenotypes also impact the pre- and post-operative parameters and clinical outcomes.

Previous studies have reported residual intra-articular varus, particularly following medial opening wedge HTO in cases with severe varus deformity [18]. Insufficient correction, inadequate soft-tissue balancing, or postoperative loss of correction have been attributed to patient-specific factors such as obesity or a preoperative JLCA greater than 6° [18,27]. However, recent studies only identified poor outcomes in patients with preoperative JLCA exceeding 6° and undergoing isolated medial opening HTO [28,29,30,31]. In our study, all preoperative JLCA values were included. In some cases, only a medial opening HTO was performed, and soft-tissue balancing was carried out based on the individual surgeon’s preference. These factors may help explain the discrepancies between the pre- and postoperative values in our cohort and the differences compared to previously reported outcomes in the literature.

This study had limitations: The clinical correlations of pre- and postoperative phenotypes were not performed, and therefore the role of the radiographic changes on functional outcomes based on the phenotype classification remains unclear. The sample size, although carefully selected, may be limited and potentially affect the statistical power of this study. Differences in surgeon experience and surgical techniques can affect radiological outcomes and cannot be entirely ruled out. The use of AI demonstrated reliable results and contributes highly to the reproducible analysis of large data sets. However, manual re-measurement of erroneous data highlights potential limitations or variations in the automated measurements. CPAK and phenotype classification were evaluated on the coronal plane. Therefore, rotational deformities of the lower extremities may influence the classification outcome.

To our knowledge, this is the first study to investigate pre- and postoperative phenotypical differences across both femoral and tibial osteotomies using CPAK and Hirschmann classifications. The findings contribute to a growing understanding of phenotype-based and personalised planning. Recent TKA trends support personalised strategies, particularly in complex deformities [32,33]. However, correlations between phenotype shifts and patient satisfaction remain an area for future research.

## 5. Conclusions

This study demonstrates a heterogeneous distribution of CPAK phenotypes following osteotomy around the knee, with postoperative alignment influenced by gender and BMI. CPAK type VI predominated after HTO in varus knees, while type V was common following DFO in valgus knees. Notably, CPAK types II, IV, and V often remained unchanged postoperatively. These findings highlight the importance of phenotype-specific planning and expose limitations of the CPAK classification in accounting for patient-specific deformities. Future studies should focus on refining classification systems to better guide surgical decision-making.

## Figures and Tables

**Table 1:** Patient demographics. Age and BMI are presented as median (minimum to maximum). Proc: procedures, Pat: patients, BMI: Body Mass Index.

Patient Demographics					
	Total	Medial Opening HTO	Medial Closing HTO	Medial Closing DFO	Lateral Closing DFO
Proc/Pat	214/188	130/115	15/14	54/44	15/15
Sex (m/f)	115/73	85/30	5/9	15/29	10/5
Age (years)	42 [14 to 65]	45 [15 to 65]	27 [16 to 53]	33.5 [14 to 57]	45 [21 to 53]
BMI (kg/m^2^)	26.8 [17.7 to 39.8]	27.0 [18.0 to 37.8]	28.4 [21.2 to 38.0]	26.7 [18.3 to 39.8]	28.9 [23.4 to 38.5]

**Table 2:** Pre- and postoperative alignment distribution sorted by HKA. Numbers are absolute, frequencies in parentheses. HTO: high tibial osteotomy, DFO: distal femoral osteotomy, HKA: hip-knee-ankle angle, Pre: preoperative, Post: postoperative.

Osteotomies Around the Knee: n = 214										
	Total		Medial Opening HTO		Medial Closing HTO		Medial Closing DFO		Lateral Closing DFO	
**Alignment (by HKA)**										
	Pre	Post	Pre	Post	Pre	Post	Pre	Post	Pre	Post
Varus knee (<−3°)	136 (63.6%)	16 (7.4%)	122 (93.8%)	1 (0.8%)	-	2 (13.3%)	-	12 (22.2%)	14 (93.3%)	1 (6.7%)
Neutral (>−3°; <3°)	16 (7.4%)	157 (73.4%)	8 (6.2%)	94 (72.3%)	2 (13.3%)	11 (73.3%)	5 (9.3%)	40 (74.1%)	1 (0.7%)	12 (80.0%)
Valgus knee (>3)	62 (30.0%)	41 (19.2%)	-	35 (26.9%)	13 (86.7%)	2 (13.3%)	49 (90.7%)	2 (3.7%)	-	2 (13.3%)

**Table 3:** Pre- and postoperative radiographic measurements of all long leg radiographs (LLRs). Values for radiographic measurements are presented as mean ± standard deviation. HTO: high tibial osteotomy, DFO: distal femoral osteotomy, Pre: preoperative, Post: postoperative, HKA: hip-knee-ankle angle, MAD: mechanical axis deviation, mLPFA: lateral proximal femoral angle, mLDFA: lateral distal femoral angle, mMPTA: mechanical medial proximal tibial angle, mLDTA: lateral distal tibial angle, JLCA: joint line convergence angle, aHKA: arithmetic HKA, JLO: joint line obliquity.

Pre- and Postoperative Radiographic Parameters										
	Total		Medial Opening HTO		Medial Closing HTO		Medial Closing DFO		Lateral Closing DFO	
	Pre	Post	Pre	Post	Pre	Post	Pre	Post	Pre	Post
HKA (°)	−2.4 ± 6.7	0.7 ± 2.8	−6.4 ± 2.5	1.6 ± 2.3	5.2 ± 3.1	0.3 ± 2.7	6.7 ± 3.4	−1.3 ± 2.7	−7.5 ± 3.4	0.7 ± 3.2
MAD (mm)	8.7 ± 23.7	−2.6 ± 9.9	22.8 ± 9.1	−5.4 ± 8.2	−18.2 ± 10.9	−0.7 ± 9.8	−23.1 ± 12.3	3.7 ± 10.0	28.2 ± 13.6	−3.2 ± 11.8
mLPFA (°)	87.6 ± 7.5	86.7 ± 5.9	88.2 ± 5.5	86.9 ± 5.8	88.1 ± 5.6	88.0 ± 5.4	85.3 ± 11.5	86.3 ± 6.0	89.2 ± 3.7	85.0 ± 6.0
mLDFA (°)	87.2 ± 3.5	87.7 ± 2.9	88.4 ± 1.8	87.5 ± 2.0	86.1 ± 2.1	85.1 ± 2.4	83.3 ± 2.7	89.9 ± 3.3	92.6 ± 3.8	84.5 ± 3.4
mMPTA (°)	86.4 ± 4.2	90.1 ± 3.3	83.9. ± 2.9	91.1 ± 3.3	93.0 ± 1.9	86.1 ± 1.9	90.0 ± 2.4	89.4 ± 2.6	88.1 ± 1.6	88.4 ± 2.0
mLDTA (°)	87.5 ± 3.8	85.9 ± 4.2	87.9 ± 3.8	86.2 ± 4.0	85.8 ± 2.9	85.0 ± 3.0	86.9 ± 3.7	85.1 ± 4.4	87.9 ± 3.7	86.3 ± 4.9
JLCA (°)	−1.6 ± 2.0	−1.6 ± 2.0	−2.0 ± 1.7	−2.1 ± 1.8	−1.8 ± 1.3	−0.6 ± 0.9	−0.2 ± 2.0	−0.6 ± 2.0	−3.1 ± 2.0	−2.9 ± 1.3
aHKA (°)	−0.8 ± 6.1	2.4 ± 3.7	−4.5 ± 2.9	3.6 ± 3.2	6.9 ± 3.1	1.0 ± 2.4	6.7 ± 3.3	−0.5 ± 3.2	−4.4 ± 3.7	3.8 ± 4.0
JLO (°)	173.6 ± 4.6	177.9 ± 5.0	172.3 ± 3.8	178.6 ± 4.4	179.1 ± 2.4	171.1 ± 3.1	173.3 ± 3.9	179.3 ± 5.0	180.7 ± 4.6	172.9 ± 3.8

**Table 4:** Distribution of postoperative CPAK types by preoperative CPAK type and osteotomy type. CPAK types are sorted by aHKA (aHKA = mMPTA − mLDFA; varus: aHKA < −2°; neutral: aHKA = 0° ± 2°; valgus: aHKA > 2°). Numbers are absolute, frequencies in parentheses. Greyscale: dark grey ≥ 30%, light grey ≥ 10%. CPAK: coronal plane alignment of the knee, HTO: high tibial osteotomy, DFO: distal femoral osteotomy.

			Postoperative CPAK								
			I	II	III	IV	V	VI	VII	VIII	IX
			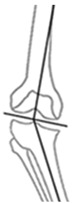	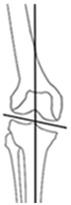	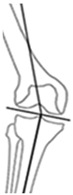	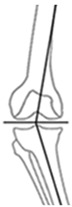	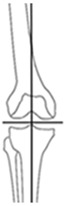	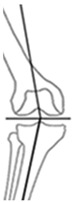	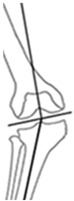	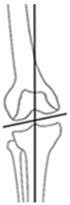	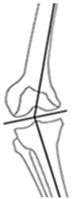
**Preoperative CPAK**	**HTO**	**I** 96 (66.2%)	2 (2.1%)	18 (18.7%)	15 (15.6%)	2 (2.1%)	14 (14.6%)	34 (4.6%)	-	2 (2.1%)	9 (9.4%)
		**II** 21 (14.5%)	-	2 (9.5%)	5 (23.8%)	1 (4.8%)	-	13 (61.9%)	-	-	-
		**III** 3 (2.1%)	-	-	2 (66.7%)	-	-	1 (33.3%)	-	-	-
		**IV** 6 (4.1%)	-	-	-	-	1 (16.7%)	3 (50.0%)	-	-	2 (33.3%)
		**V** 6 (4.1%)	-	-	-	-	-	4 (66.7%)	-	-	2 (33.3%)
		**VI** 11 (7.6%)	1 (9.1%)	4 (36.4%)	6 (54.5%)	-	-	-	-	-	-
		**IX** 2 (1.4%)	1 (50.0%)	-	-	-	1 (50.0%)	-	-	-	-
		**Total** 145	4 (2.8%)	24 (16.5%)	28 (19.3%)	3 (2.1%)	16 (11.0%)	55 (37.9%)	-	2 (1.4%)	13 (9.0%)
	**DFO**	**I**1 (1.4%)	-	-	1 (100%)	-	-	-	-	-	-
		**II** 3 (4.4%)	-	1 (33.3%)	1 (33.3%)	1 (33.3%)	-	-	-	-	-
		**III** 44 (63.8%)	3 (6.8%)	5 (11.4%)	4 (9.1%)	8 (18.2%)	12 (27.3%)	5 (11.4%)	5 (11.4%)	1 (2.3%)	1 (2.3%)
		**IV** 8 (11.6%)	-	2 (25.0%)	6 (75.0%)	-	-	-	-	-	-
		**V** 2 (2.9%)	-	-	2 (100%)	-	-	-	-	-	-
		**VI** 7 (10.1%)	-	-	-	1 (14.3%)	1 (14.3%)	-	2 (28.6%)	2 (28.6%)	1 (14.3%)
		**VII** 4 (5.8%)	-	-	2 (50.0%)	-	-	1 (25.0%)	1 (25.0%)	-	-
		**Total** 69	4 (5.5%)	9 (12.3%)	17 (23.3%)	10 (13.7%)	14 (19.2%)	6 (8.2%)	8 (11.0%)	3 (4.1%)	2 (2.7%)

**Table 5:** Distribution of femoral- and tibial morphotype grouped by pre- and postoperative osteotomy type (HTO and DFO) and subdivided into the preoperative alignment by the HKA (varus, neutral, valgus). Classification was performed according to mechanical lateral distal femur angle (mLDFA) and mechanical medial tibial angle (mMPTA). Numbers are absolute, frequencies in parentheses. HTO: high tibial osteotomy, DFO: distal femoral osteotomy, VAR: varus, NEU: neutral, VAL: valgus.

145		Preoperative HTO: n = 132					Postoperative HTO: n = 128						
		VAR _mLDFA_6°	VAR _mLDFA_3°	NEU _mLDFA_0°	VAL _mLDFA_3°	VAL _mLDFA_6°			VAR_mLDFA_6°	VAR_mLDFA_3°	NEU_mLDFA_0°	VAL_mLDFA_3°	VAL_mLDFA_6°
**Varus (85.6%)**	**VAR_mMPTA_6°**	1 (3.6%)	8 (28.6%)	17 (60.7%)	2 (7.1%)	-	**Varus (2.3%)**	**VAR_mMPTA_6°**	-	-	-	-	-
	**VAR_mMPTA_3°**	6 (10.5%)	23 (40.4%)	26 (45.6%)	2 (3.5%)	-		**VAR_mMPTA_3°**	-	-	-	1 (100%)	-
	**NEU_mMPTA_0°**	1 (3.9%)	18 (69.2%)	7 (26.9%)	-	-		**NEU_mMPTA_0°**	-	-	1 (100%)	-	-
	**VAL_mMPTA_3°**	-	1 (50.0%)	1 (50.0%)	-	-		**VAL_mMPTA_3°**	-	-	-	-	1 (100%)
	**VAL_mMPTA_6°**	-	-	-	-	-		**VAL_mMPTA_6°**	-	-	-	-	-
**Neutral (7.6%)**	**VAR_mMPTA_6°**	-	-	-	-	-	**Neutral (79.7%)**	**VAR_mMPTA_6°**	-	-	-	1 (100%)	-
	**VAR_mMPTA_3°**	-	-	1 (100%)	-	-		**VAR_mMPTA_3°**	-	-	4 (33.3%)	7 (58.3%)	1 (8.3%)
	**NEU_mMPTA_0°**	-	1 (25.0%)	2 (50.0%)	1 (25.0%)	-		**NEU_mMPTA_0°**	-	-	12 (48.0%)	13 (52.0%)	-
	**VAL_mMPTA_3°**	-	1 (25.0%)	3 (75.0%)	-	-		**VAL_mMPTA_3°**	-	-	17 (53.1%)	15 (46.9%)	-
	**VAL_mMPTA_6°**	-	1 (100%)	-	-	-		**VAL_mMPTA_6°**	-	-	12 (37.5%)	19 (59.4%)	1 (3.1%)
**Valgus (6.8%)**	**VAR_mMPTA_6°**	-	-	-	-	-	**Valgus (18.0%)**	**VAR_mMPTA_6°**	-	-	-	-	-
	**VAR_mMPTA_3°**	-	-	-	-	-		**VAR_mMPTA_3°**	-	-	-	-	-
	**NEU_mMPTA_0°**	-	-	-	-	-		**NEU_mMPTA_0°**	-	-	-	-	1 (100%)
	**VAL_mMPTA_3°**	-	-	-	1 (100%)	-		**VAL_mMPTA_3°**	-	-	5 (50.0%)	5 (50.0%)	-
	**VAL_mMPTA_6°**	-	-	6 (75.0%)	2 (25.0%)	-		**VAL_mMPTA_6°**	-	-	6 (50.0%)	6 (50.0%)	-
		**Preoperative DFO: n = 60**							**Postoperative DFO: n = 63**				
		**VAR_mLDFA_6°**	**VAR_mLDFA_3°**	**NEU_mLDFA_0°**	**VAL_mLDFA_3°**	**VAL_mLDFA_6°**			**VAR_mLDFA_6°**	**VAR_mLDFA_3°**	**NEU_mLDFA_0°**	**VAL_mLDFA_3°**	**VAL_mLDFA_6°**
**Varus (20.0%)**	**VAR_mMPTA_6°**	-	-	-	-	-	**Varus (15.9%)**	**VAR_mMPTA_6°**	-	-	-	-	-
	**VAR_mMPTA_3°**	1 (100%)	-	-	-	-		**VAR_mMPTA_3°**	-	-	1 (50.0%)	-	1 (50.0%)
	**NEU_mMPTA_0°**	4 (57.1%)	3 (42.9%)	-	-	-		**NEU_mMPTA_0°**	-	-	-	1 (25.0%)	3 (75.0%)
	**VAL_mMPTA_3°**	2 (50.0%)	2 (50.0%)	-	-	-		**VAL_mMPTA_3°**	-	-	-	1 (25.0%)	3 (75.0%)
	**VAL_mMPTA_6°**	-	-	-	-	-		**VAL_mMPTA_6°**	-	-	-	-	-
**Neutral (10.0 %)**	**VAR_mMPTA_6°**	-	-	-	-	-	**Neutral (79.4%)**	**VAR_mMPTA_6°**	-	-	-	-	-
	**VAR_mMPTA_3°**	-	-	-	-	-		**VAR_mMPTA_3°**	-	-	-	1 (33.3)	2 (66.7%)
	**NEU_mMPTA_0°**	-	-	3 (60.0%)	2 (40.0%)	-		**NEU_mMPTA_0°**	-	-	3 (15.0%)	16 (80.0%)	1 (5.0%)
	**VAL_mMPTA_3°**	-	-	-	1 (100%)	-		**VAL_mMPTA_3°**	-	-	8 (34.8%)	12 (52.2%)	3 (13.0%)
	**VAL_mMPTA_6°**	-	-	-	-	-		**VAL_mMPTA_6°**	-	-	1 (25.0%)	2 (50.0%)	1 (25.0%)
**Valgus (70.0%)**	**VAR_mMPTA_6°**	-	-	-	-	-	**Valgus (4.5%)**	**VAR_mMPTA_6°**	-	-	-	-	-
	**VAR_mMPTA_3°**	-	-	-	-	1 (100%)		**VAR_mMPTA_3°**	-	-	-	-	1 (100%)
	**NEU_mMPTA_0°**	-	-	-	3 (50.0%)	3 (50.0%)		**NEU_mMPTA_0°**	-	-	-	-	-
	**VAL_mMPTA_3°**	1 (3.6%)	-	2 (7.1%)	19 (67.9%)	6 (21.4%)		**VAL_mMPTA_3°**	-	-	-	-	2 (100%)
	**VAL_mMPTA_6°**	-	-	2 (28.6%)	5 (71.4%)	-		**VAL_mMPTA_6°**	-	-	-	-	-

## Data Availability

The data that support the findings of this study are available from the corresponding author upon reasonable request.

## References

[1] Micicoi G., Grasso F., Kley K., Favreau H., Khakha R., Ehlinger M., Jacquet C., Ollivier M. (2021). Osteotomy around the knee is planned toward an anatomical bone correction in less than half of patients. Orthop. Traumatol. Surg. Res..

[2] Dawson M.J., Ollivier M., Menetrey J., Beaufils P. (2022). Osteotomy around the painful degenerative varus knee: A 2022 ESSKA formal consensus. Knee Surg. Sports Traumatol. Arthrosc..

[3] Ramazanian T., Yan S., Rouzrokh P., Wyles C.C., O Byrne T.J., Taunton M.J., Maradit Kremers H. (2022). Distribution and Correlates of Hip-Knee-Ankle Angle in Early Osteoarthritis and Preoperative Total Knee Arthroplasty Patients. J. Arthroplast..

[4] Cooke D., Scudamore A., Li J., Wyss U., Bryant T., Costigan P. (1997). Axial lower-limb alignment: Comparison of knee geometry in normal volunteers and osteoarthritis patients. Osteoarthr. Cartil..

[5] Radler C., Antonietti G., Ganger R., Grill F. (2011). Recurrence of axial malalignment after surgical correction in congenital femoral deficiency and fibular hemimelia. Int. Orthop..

[6] Micicoi G., Jacquet C., Sharma A., LiArno S., Faizan A., Kley K., Parratte S., Ollivier M. (2021). Neutral alignment resulting from tibial vara and opposite femoral valgus is the main morphologic pattern in healthy middle-aged patients: An exploration of a 3D-CT database. Knee Surg. Sports Traumatol. Arthrosc..

[7] Nakayama H., Schröter S., Yamamoto C., Iseki T., Kanto R., Kurosaka K., Kambara S., Yoshiya S., Higa M. (2018). Large correction in opening wedge high tibial osteotomy with resultant joint-line obliquity induces excessive shear stress on the articular cartilage. Knee Surg. Sports Traumatol. Arthrosc..

[8] MacDessi S.J., Griffiths-Jones W., Harris I.A., Bellemans J., Chen D.B. (2021). Coronal Plane Alignment of the Knee (CPAK) classification. Bone Jt. J..

[9] Hirschmann M.T., Moser L.B., Amsler F., Behrend H., Leclerq V., Hess S. (2019). Functional knee phenotypes: A novel classification for phenotyping the coronal lower limb alignment based on the native alignment in young non-osteoarthritic patients. Knee Surg. Sports Traumatol. Arthrosc..

[10] Huber S., Mitterer J.A., Vallant S.M., Simon S., Hanak-Hammerl F., Schwarz G.M., Klasan A., Hofstaetter J.G. (2023). Gender-specific distribution of knee morphology according to CPAK and functional phenotype classification: Analysis of 8739 osteoarthritic knees prior to total knee arthroplasty using artificial intelligence. Knee Surg. Sports Traumatol. Arthrosc..

[11] Franceschetti E., Campi S., Giurazza G., Tanzilli A., Gregori P., Laudisio A., Hirschmann M.T., Samuelsson K., Papalia R. (2024). Mechanically aligned total knee arthroplasty does not yield uniform outcomes across all coronal plane alignment of the knee (CPAK) phenotypes. Knee Surg. Sports Traumatol. Arthrosc..

[12] Paley D. (2002). Principles of Deformity Correction.

[13] Mitterer J.A., Huber S., Schwarz G.M., Simon S., Pallamar M., Kissler F., Frank B.J.H., Hofstaetter J.G. (2023). Fully automated assessment of the knee alignment on long leg radiographs following corrective knee osteotomies in patients with valgus or varus deformities. Arch. Orthop. Trauma Surg..

[14] (2014). Steve Bogart SankeyMATIC. https://sankeymatic.com/build/.

[15] Cohen J. (2013). Statistical Power Analysis for the Behavioral Sciences.

[16] Tseng T.H., Wang H.Y., Tzeng S.C., Hsu K.H., Wang J.H. (2022). Knee-ankle joint line angle: A significant contributor to high-degree knee joint line obliquity in medial opening wedge high tibial osteotomy. J. Orthop. Surg. Res..

[17] Mullaji A., Bhoskar R., Singh A., Haidermota M. (2022). Valgus arthritic knees can be classified into nine phenotypes. Knee Surg. Sports Traumatol. Arthrosc..

[18] Ji W., Luo C., Zhan Y., Xie X., He Q., Zhang B. (2019). A residual intra-articular varus after medial opening wedge high tibial osteotomy (HTO) for varus osteoarthritis of the knee. Arch. Orthop. Trauma Surg..

[19] Charre D., An J.S., Khakha R., Kley K., Şahbat Y., Ollivier M. (2024). ‘One millimetre equals one degree’ is a major source of inaccuracy in planning osteotomies around the knee for metaphyseal deformities compared to the digital planning. Knee Surg. Sports Traumatol. Arthrosc..

[20] Kley K., Bin Abd Razak H.R., Khakha R.S., Wilson A.J., van Heerwaarden R., Ollivier M. (2021). Soft-Tissue Management and Neurovascular Protection During Opening-Wedge High Tibial Osteotomy. Arthrosc. Tech..

[21] Ollivier M., Claes S., Mabrouk A., Elson D., Espejo-Reina A., Predescu V., Schröter S., Van heerwarden R., Menetrey J., Beaufils P. (2024). Surgical strategy and complication management of osteotomy around the painful degenerative varus knee: ESSKA Formal Consensus Part II. Knee Surg. Sports Traumatol. Arthrosc..

[22] Dawson M., Elson D., Claes S., Predescu V., Khakha R., Espejo-Reina A., Schröter S., van Heerwarden R., Menetrey J., Beaufils P. (2024). Osteotomy around the painful degenerative varus knee has broader indications than conventionally described but must follow a strict planning process: ESSKA Formal Consensus Part I. Knee Surg. Sports Traumatol. Arthrosc..

[23] Liu L.M., Lei K., Du D., Lin Y., Pan Z., Guo L. (2024). Functional knee phenotypes appear to be more suitable for the Chinese OA population compared with CPAK classification: A study based on 3D CT reconstruction models. Knee Surgery, Sport. Traumatol. Arthrosc..

[24] An J.S., Jacquet C., Loddo G., Mabrouk A., Koga H., Argenson J.N., Ollivier M. (2024). Deformity in valgus knee malalignment is not only in the femur but also in tibia or both, based on demographic and morphological analysis before and after knee osteotomies. Knee Surg. Sports Traumatol. Arthrosc..

[25] Sakai M., Akasaki Y., Akiyama T., Horikawa T., Okazaki K., Hamai S., Tsushima H., Kawahara S., Kurakazu I., Kubota K. (2023). Similar short-term KOOS between open-wedge high tibial osteotomy and total knee arthroplasty in patients over age 60: A propensity score–matched cohort study. Mod. Rheumatol..

[26] Akçaalan S., Akkaya M., Dogan M., Valdivielso A.A., Zeiton M.A., Mohammad H.R., Sangaletti R., Benazzo F., Kara S., Gehrke T. (2023). Do age, gender, and region affect tibial slope? A multi-center study. Arch. Orthop. Trauma Surg..

[27] Brouwer G.M., Van Tol A.W., Bergink A.P., Belo J.N., Bernsen R.M.D., Reijman M., Pols H.A.P., Bierma-Zeinstra S.M.A. (2007). Association between valgus and varus alignment and the development and progression of radiographic osteoarthritis of the knee. Arthritis Rheum..

[28] Harris C., Nadeem F., Hargreaves M., Campbell C., Momaya A., Casp A. (2024). Obesity does not impact complications and conversion to total knee arthroplasty after high tibial osteotomy: A systematic review. Knee Surg. Sports Traumatol. Arthrosc..

[29] Siboni R., Beaufils P., Boisrenoult P., Steltzlen C., Pujol N. (2018). Opening-wedge high tibial osteotomy without bone grafting in severe varus osteoarthritic knee. Rate and risk factors of non-union in 41 cases. Orthop. Traumatol. Surg. Res..

[30] Na Y.G., Lee B.K., Choi J.U., Lee B.H., Sim J.A. (2021). Change of joint-line convergence angle should be considered for accurate alignment correction in high tibial osteotomy. Knee Surg. Relat. Res..

[31] Sohn S., Koh I.J., Kim M.S., In Y. (2022). Risk factors and preventive strategy for excessive coronal inclination of tibial plateau following medial opening-wedge high tibial osteotomy. Arch. Orthop. Trauma Surg..

[32] Begum F.A., Kayani B., Magan A.A., Chang J.S., Haddad F.S. (2021). Current concepts in total knee arthroplasty. Bone Jt. Open.

[33] Cherian J.J., Kapadia B.H., Banerjee S., Jauregui J.J., Issa K., Mont M.A. (2014). Mechanical, anatomical, and kinematic axis in TKA: Concepts and practical applications. Curr. Rev. Musculoskelet. Med..

